# Inhibition of *Col6a5* Improve Lipid Metabolism Disorder in Dihydrotestosterone-Induced Hyperandrogenic Mice

**DOI:** 10.3389/fcell.2021.669189

**Published:** 2021-05-24

**Authors:** Li-Feng Sun, Ya-Li Yang, Mei-Yue Wang, Hua-Shan Zhao, Tian-xia Xiao, Meng-Xia Li, Bao-Bei Wang, Chen Huang, Pei-Gen Ren, Jian V. Zhang

**Affiliations:** ^1^Center for Energy Metabolism and Reproduction, Shenzhen Institutes of Advanced Technology, Chinese Academy of Sciences, Shenzhen, China; ^2^Shenzhen College of Advanced Technology, University of Chinese Academy of Sciences, Shenzhen, China; ^3^Department of Clinical Pharmacy and Translational Medicine, School of Pharmacy and Biomedicine, Shenzhen Institutes of Advanced Technology, Chinese Academy of Sciences, Shenzhen, China

**Keywords:** dihydrotestosterone, ovary, gonad fat, **Col6a5**, hypertrophy, lipid metabolism

## Abstract

Hyperandrogenism is a key pathological feature of polycystic ovarian syndrome (PCOS). Excess androgen can lead to PCOS-like cell hypertrophy in the ovaries and adipose tissue of rodents. Here, we established a dihydrotestosterone (DHT)-induced hyperandrogenic mouse model to analyze the differences in gene expression and signaling pathways of the ovaries and gonad fat pads of mice treated with or without DHT by RNA microarray analysis. From the results, we focused on the overlapping differentially expressed gene—*Col6a5*—and the major differentially enriched signaling pathway—lipid metabolism. We employed DHT-induced mouse ovarian stromal cell, adipogenic 3T3-L1 cell and hepatic cell line NCTC1469 models to investigate whether androgens directly mediate lipid accumulation and hypertrophy. We found that DHT increased lipid droplet accumulation in ovarian stromal cells and adipogenic 3T3-L1 cells but not NCTC1469 cells. DHT significantly altered stromal cell cholesterol metabolism and steroidogenesis, as indicated by changes in cholesterol levels and the expression of related genes, but these effects were not observed in 3T3-L1 cells. Moreover, *Col6a5* expression was significantly increased in ovaries and gonadal fat pads of DHT-treated mice, and *Col6a5* inhibition alleviated DHT-induced excess lipid accumulation and hypertrophy of ovarian stromal cells and adipogenic 3T3-L1 cells, even improved lipid metabolism in overnourished NCTC1469 cells. Our results indicate that *Col6a5* plays important roles in the pathogenesis of DHT-induced lipid metabolism disorder and the hypertrophy of ovarian stromal cells and adipocytes.

## Introduction

Hyperandrogenism is a complex hormonal disorder with a high incidence rate and a wide range of manifestations. The most common disease associated with hyperandrogenism is polycystic ovary syndrome (PCOS), which is characterized by metabolic and reproductive dysfunction and affects 10–15% of women of childbearing age (Gorry et al., 2006; Fauser et al., 2012). Hyperandrogenic rodent models induced with dihydrotestosterone (DHT), a non-aromatizable androgen, exhibit PCOS-like phenotypes, including irregular cycles, polycystic ovaries, insulin resistance and increased body weight (Manneras et al., 2007; Caldwell et al., 2014). In addition, DHT disrupts pancreatic beta cell function (Wang et al., 2015), cardiac metabolism (Tepavcevic et al., 2014, 2015), and circadian rhythm (Mereness et al., 2015) and leads to hypertension (Hoang et al., 2015) and endothelial dysfunction (Keller et al., 2011).

DHT-treated hyperandrogenic rodents show increased levels of serum cholesterol and triglycerides, which are components of lipid metabolism (Manneras et al., 2007; Salilew-Wondim et al., 2015); increased fat mass based on the enlargement and increased number of adipocytes (Manneras et al., 2007; van Houten et al., 2012; Wang et al., 2015); and pronounced thickening of the theca cell layer and hyperplasia of stroma (Hillier, 1979; Manneras et al., 2007). However, it remains unclear whether cell hypertrophy and lipid accumulation occur simultaneously in ovarian cells as in adipose tissue. Hence, we focused on the mechanism and correlation between cell hypertrophy and lipid accumulation in the ovaries and gonadal fat pads to explore and systematically analyze lipid metabolism-related gene expression and signaling pathways by RNA microarray analysis of DHT-treated mouse ovaries and gonadal fat pads.

Based on the analysis of differentially expressed genes, we selected collagen type VI alpha 5 (*Col6a5*), which was overexpressed in both the hypertrophic ovaries and gonadal fat pats of DHT-treated mice. Collagen is one of the main components of the extracellular matrix (ECM) that plays a role in cell hypertrophy. Many studies have reported that hepatic lipid accumulation is associated with an increase in ECM collagen proteins (Sanyal et al., 2001; Puri et al., 2007; Wada et al., 2013). In addition, integrin, a major collagen receptor expressed on various cell types (Volpes et al., 1991), has been shown to regulate lipid accumulation (Pankov et al., 2005). However, the role of collagen or *Col6a5* in the process of ovarian cell hypertrophy has not yet been reported and deserves further study.

It is generally believed that hyperandrogenism can induce cell hypertrophy (Battaglia et al., 2012; Echiburu et al., 2018). However, whether the disorder originates from the tissue itself or manifests elsewhere and then induces downstream ramifications remains unknown. We hypothesize that androgen acts directly on ovarian cells and adipocytes, leading to lipid metabolism disorder and hypertrophy, which can be ameliorated by *Col6a5* suppression. Therefore, in this study, we established a DHT-induced hyperandrogenic mouse model (Manneras et al., 2007; van Houten et al., 2012) and hyperandrogenic cell models to explore the effects of androgen and the mechanism of *Col6a5*. Our findings highlight the importance of *Col6a5*-mediated androgen actions in lipid metabolism disorder and the process of ovarian stromal cell and adipocyte hypertrophy.

## Materials and Methods

### Ethics

The animal study was reviewed and approved by The Committee on the Use of Live Animals for Teaching and Research, Shenzhen Institutes of Advanced Technology, Chinese Academy of Sciences. All animal experimental methods were performed in accordance with the approved guidelines and regulations.

### Animal Experiments

C57BL/6J wild-type female mice were purchased from Beijing Vital River Laboratory Animal Technology Co., Ltd. and acclimated to housing conditions for 1 week at a temperature of 22.0 ± 1°C, humidity of 40–60% and a 12-h light/dark cycle with fresh food and water available *ad libitum.*

At postnatal day 25, mice of comparable body weight were randomly divided into four treatment group (CTRL, DHT, DHT + NC-LV, DHT + *Col6a5*-LV. *n* = 6 per subgroup). The mice from DHT, DHT + NC-LV, DHT + *Col6a5*-LV groups were implanted subcutaneously (sc) with 1.7-cm silicone capsules (size: 1.57 mm inner diameter (ID) × 3.18 mm outer diameter (OD), Dow Corning, United States) containing 5 mg of DHT powder. Control mice (CTRL) received empty capsules (van Houten et al., 2012). *Col6a5*-LV is a recombinant *Col6a5*-CRISPR/Cas9 lentivirus, and NC-LV is a control lentivirus. The lentiviruses were delivered by hydrodynamic tail vein injection from the first day of DHT treatment (106 lentivirus particles/mouse/per week) (Chen and Calvisi, 2014).

Mice were sacrificed at the end of the 60-days treatment period. To determine the stage of the estrous cycle, daily vaginal smears were taken and examined 2 weeks before the animals were killed. The body weight of each mouse was measured every 10 days. In addition, at the end of the 60-days treatment period, blood samples were collected by orbital puncture after the mice were anesthetized with isoflurane, and tissues (ovaries, gonadal fat pads) were isolated and fixed overnight in 4% paraformaldehyde (PFA) or soaked in RNAiso Plus (−80°C cryopreservation).

### Intraperitoneal Glucose Tolerance Test (IPGTT)

For the IPGTT, female mice were fasted for 16 h with free access to drinking water and intraperitoneally (i.p.) injected with D-glucose (2.0 g/kg body weight). Blood glucose levels were measured before and 15, 30, 60, 90, and 120 min after i.p. glucose injection by using an Accu-Chek glucose monitor (Roche Diagnostics Australia Pty. Ltd., Australia) (*n* = 6 per group).

### RNA Analysis by Quantitative PCR (qPCR)

Total RNA from tissues and cells was extracted using RNAiso Plus and subjected to qPCR analysis. RNA samples were reverse transcribed into cDNA according to the manufacturer’s instructions (Toyobo, Osaka, Japan). The PCR mixtures contained 10 μl of SYBR Premix Ex Taq II (Toyobo), 1 μl of each primer, 1 μl of cDNA, and 7 μl of DNase-free water to a final volume of 20 μl. The cycle conditions were 1 min at 95°C, followed by 45 cycles at 95°C for 15 s, 65°C for 30 s, and 72°C for 1 min. The primers were designed on the basis of the published sequences. The RNA levels were calculated by the 2^−^ΔΔ^*CT*^ method, where CT is the cycle threshold (Livak and Schmittgen, 2001; Table 1 contains the primer sequences).

**TABLE 1:** Primer sequences.

Genes name	Upstream primer sequences (5’–3’)	Downstream primer sequences (5’–3’)
β*actin*	GTGACGTTGACATCCGTAAAGA	GCCGGACTCATCGTACTCC
*StAR*	CCGGGTGGATGGGTCAA	CACCTCTCCCTGCTGGATGTA
*P450scc*	CCATCAGATGCAGAGTTTCCAA	TGAGAAGAGTATCGACGCATCCT
*3*β*-HSD*	GGAGGCCTGTGTTCAAGCAA	GGCCCTGCAACATCAACTG
*17*β*-HSD*	TTGTTTGGGCCGCTAGAAG	CACCCACAGCGTTCAATTCA
*Cd36*	AGATGACGTGGCAAAGAACAG	CCTTGGCTAGATAACGAACTCTG
*ADD1*	GGAACATGGCACCAGACCTTC	AAGGCAGGACTCTGTAGAATCA
*MTTP*	ATACAAGCTCACGTACTCCACT	TCTCTGTTGACCCGCATTTTC
*Sf1*	AAAATACGACGACTACCACCAC	GGTGACAAAGTTAGAAGGGTCCA
*DAX1*	GGTCCCTCTTGTACCGCTG	TCTTCTCCGCAGAAACAACAG
*GATA6*	TTGCTCCGGTAACAGCAGTG	GTGGTCGCTTGTGTAGAAGGA
*Twist2*	CGCTACAGCAAGAAATCGAGC	GCTGAGCTTGTCAGAGGGG
*Chop*	CTGGAAGCCTGGTATGAGGAT	CAGGGTCAAGAGTAGTGAAGGT
*Col6a5*	CCAAACATGACACGGATCATCA	GGAACTGTCTTATCAACGTGGT

### Gene Expression Analysis

Ovaries and gonadal fat pads from the Ctrl and DHT treatment groups were sent to Genminix Informatics Ltd., Shanghai, China, for gene expression analysis via the Affymetrix GeneChip^®^ Mouse Transcriptome Assay 1.0 array (*n* = 3 per group). The signatures and abundances of RNAs can be found in National Center for Biotechnology Information, Gene Expression Omnibus (NCBI, GEO) under series number GSE171431.

Differential expression between the comparison groups was determined using online tools from the Gene-Cloud of Biotechnology Information (GCBI)^1^, based on the SAM method for at least three samples per group. GO and annotation analysis of disordered genes was performed using Metascape^2^, based on Fisher’s exact test, which was represented in a 2 × 2 contingency table. mRNA transcripts were analyzed after using GCBI to filter out genes with detection rates below 50% in the overall expression profile. The mRNAs between the comparison groups exhibited expression fold-changes ≥ 2 and *p*-values ≤ 0.01, which were considered significant (Huang et al., 2018). Gene set enrichment analysis (GSEA) was conducted on the desktop version of the GSEA software (v.4.1.0).

### Morphological Analysis

Ovaries and gonadal fat pads were dehydrated with a graded alcohol series and embedded in paraffin. Serial 5-μm sections were stained with hematoxylin and counterstained with eosin. The cell size was measured with Image-Pro Plus 6.0 software. Briefly, the perimeter of the stroma area was manually drawn (Supplementary Figure 2). Then, the area was measured, and the number of cells in the area was counted. The ratio of the cell area to the cell number was calculated. Three to five fields of view were measured on each ovary to cover whole ovarian stromal cells within each section (*n* = 6 per group). The diameters of adipocytes were evaluated by automatically counting bright objects and measuring the mean diameter. Three to five fields of view were measured on each gonadal fat pad to cover adipocytes of different sizes within each section (*n* = 6 per group).

Immunohistochemistry (IHC) and Immunofluorescence (IF) were carried out on 5-μm paraffin-embedded ovaries sections. After deparaffifinization and rehydration, 3% H_2_O_2_ was used to block endogenous peroxidase activity for 30 min at room temperature. For non-specific binding inhibition, the sections were blocked with 1% BSA for 1 h and incubated overnight at 4°C with anti-*Col6a5* antibody (diluted 1:100, ThermoFisher, PA570781) and anti-PLIN2 antibody (diluted 1:100, Abcam, ab108323). After washing, the sections were incubated with HRP-Goat-anti-Rabbit antibody (diluted 1:200, Abcam, ab97051) or Alexa-488-Donkey anti- rabbit (diluted 1:200, Invitrogen, A21206) for 1 h. IHC staining was visualized using the 3,3’-Diaminobenzidine (DAB) Substrate Kit for Peroxidase (Beyotime, P0203), and the slides were counterstained with hematoxylin. For IF staining, sections were viewed under an Olympus microscope after the nuclei were stained with DAPI (diluted 1:1,000, sigma, D9542). Control sections were immunostained with non-specific IgG to check for non-specific staining.

### Triglyceride (TG) and Total Cholesterol (TC) Content

The TG and TC contents in mice ovarian stromal cells and 3T3-L1 cells were analyzed. The cells were washed with PBS, harvested by trypsinization and then resuspended in 1 ml PBS (∼10^6^ cells). The cell suspension was homogenized by sonication for 5 min. The TG and TC contents were determined using a commercial assay kit according to the manufacturer’s protocol (GPO-PAP, Nanjing Jiancheng Bioengineering Institute, Nanjing, China). The protein concentration was determined using the Pierce^TM^ BCA Protein Assay Kit.

### Stromal Cell Isolation and Culture

We used 21-day-old immature female mice for this study. The collection and purification of ovarian stromal cells were performed as described previously (Magoffin and Erickson, 1988; Duleba et al., 1997). Briefly, the ovaries were removed from the animals and dissected from the oviducts and fat under a dissecting microscope. After 60 min of collagenase digestion, stromal cells were purified using discontinuous Percoll gradient centrifugation. The stromal cells were incubated at a density of 10^4^ cells/ml. The cultures were carried out for 48 h at 37°C in an atmosphere of 5% CO_2_ humidified air in serum-free McCoy’s 5A culture medium supplemented with 1% antibiotic/antimycotic mix and 10% fetal bovine serum (FBS). All cultures were carried out in the presence of ovine luteinizing hormone (LH) (5 ng/ml).

### *Col6a5* Knockdown in Stromal Cells and NCTC1469 Cells

For transient transfection, stromal cells and NCTC1469 cells were grown to 80% confluence with complete medium and transfected with *Col6a5*-siRNA (sense 5′–3′ CCAAUUACCUGGGAAUUAATT, antisense 5′–3′ UUAAUUCCCAGGUAAUUGGTT) or negative control siRNA (NC-RNA, sense 5′–3′ UUCUCCGAACGUGUCACGUTT, antisense 5′–3′ ACGUGACACGUUCGGAGAATT) using Lipofectamine 2000 reagent (Invitrogen). At 4–6 h post-transfection, the cells were washed and incubated in conditioned medium (complete medium containing DHT or inhibitors) for 24 h. The extent of *Col6a5* knockdown with siRNA was shown in Supplementary Figure 6.

### Packaging of the Recombinant Lentiviral Vectors

The *Col6a5* sgRNA CRISPR/Cas9 All-in-One Lentivector with the target sequence TGTTTGCCCCAAACATGACA (K4947208, Applied Biological Materials (ABM) Inc., Vancouver, Canada) was cotransfected with the packaging plasmids pHelper1.0 and pHelper2.0 into 293T cells to prepare the recombinant lentiviral vector *Col6a5*-LV. Scrambled sgRNA CRISPR/Cas9 All-in-One Lentivector was used as the control lentivirus [NC-LV including the target sequence GCACTCACATCGCTACATCA (K010, ABM)]. These vectors were concentrated to 10^7^ lentivirus particles/ml. The concentration of lentivirus particles was measured with a commercial ELISA kit (Beijing Biodragon lmmunotechnologies Co., Ltd., Beijing, China).

### *Col6a5* Knockdown in 3T3-L1 Cells

3T3-L1 preadipocytes [American Type Culture Collection (ATCC)] were allowed to grow to 60% confluence in 6-well plates, and then the cells were infected with lentivirus (1 ml *Col6a5*-LV plus 1 ml complete medium). Twelve hours post-infection, the medium was replaced with fresh complete medium. Three to four days later, the infected cells were cultured with 10 μg/ml puromycin for 1 week for selection. Control cell lines were generated in the same way as described above with the control lentivirus (NC-LV). The extent of *Col6a5* knockdown with lentivirus was shown in Supplementary Figure 6.

### 3T3-L1 Induction Into Adipocytes

3T3-L1 preadipocytes were allowed to grow to 80% confluence in complete medium (high glucose Dulbecco’s modified Eagle’s medium (Gibco), 10% FBS and 1% antibiotic/antimycotic mix). Then, the 3T3-L1 preadipocytes were seeded in 6-well plates and stimulated with 0.5 mM 1-isobutyl-3-methylxanthine (IBMX), 1 mM dexamethasone, and 10 μg/ml insulin for 2 days. DMEM was then replenished with the addition of 10 μg/ml insulin for 2 days, and then the medium was replaced with DMEM without insulin for 2 days (Li et al., 2016).

### FBS-Induced Steatosis in NCTC1469 Cells

NCTC1469 cells were routinely cultured in growth medium (DMEM with 10% FBS and 1% penicillin-streptomycin solution) in a humid incubator at 37°C and 5% CO_2_. Fat-overloading induction of the cells was performed when the cells grew to 80% confluence; the growth medium was removed, and the cells were divided into two groups: the control group and the model group. The control group was cultured in growth medium, and the model group was cultured in high-FBS medium (DMEM with 50% FBS and 1% penicillin-streptomycin solution) for 24 h to induce steatosis (Liu and Huang, 2016).

### DHT Cell Treatment

For steroid treatment experiments, DHT (in 100% EtOH) at the appropriate concentration (0.1, 1, 10, and 100 μM) or EtOH vehicle was added to the culture medium (to a final EtOH concentration of 0.00001%) (Mereness et al., 2015). Stromal cells and NCTC1469 cells were treated with DHT for 24 h. 3T3-L1 cells were treated for 6 days.

### Stage-Specific Inhibition of Lipid Accumulation in Cells

To explore which stages of lipid accumulation in cells were affected by DHT, the cells were treated with stage-specific inhibitors, simvastatin (an inhibitor of cholesterol synthesis), ezetimibe (an inhibitor of cholesterol uptake), and 4-phenylbutyric acid (4-PBA, an inhibitor of endoplasmic reticulum stress). Inhibitors (in 100% EtOH) at the appropriate concentration or EtOH vehicle was added to the culture medium (to a final EtOH concentration < 0.001%). The doses were as follows: simvastatin (0.005, 0.05, and 0.5 μM), ezetimibe (0.3, 3, and 30 μM) and 4-PBA (0.01, 0.1, and 1 μM) (Yu et al., 2006; Zhang et al., 2012; Tung et al., 2015). Stromal cells and NCTC1469 cells were treated with inhibitor for 24 h, and 3T3-L1 cells were treated for 6 days.

### Oil Red O Staining

Intracellular lipid accumulation was determined by Oil Red O staining. After removal of the culture media, the cells were washed twice with PBS, fixed with 4% PFA, and stained with Oil Red O (six parts 0.6% Oil Red O dye in isopropanol and four parts water) for 30 min. After rinsing three times with distilled water, the cells were photographed under a microscope.

To quantify lipid accumulation, lipids and Oil Red O were dissolved in isopropanol, and absorbance was measured by a microplate spectrophotometer at 500 nm. The percentage of Oil Red O-stained material relative to that in control wells was calculated as the absorbance at 500 nm (sample)/the absorbance at 500 nm (control) (Li et al., 2016).

### Hormone Assay

The serum of mice treated with or without DHT were collected, and DHT levels were measured with a mouse DHT ELISA kit (Jiangsu Enzyme Biotechnology, MB-3267A). The intra- and interassay error rates among all assays were less than 10 and 15%, respectively.

### Statistical Analysis

Results are expressed as mean ± SEM. Duncan’s multiple range test for individual comparison repeated measures ANOVA was used to analyzed body weight and serum glucose. Mann-Whitney *U*-test (a non-parametric statistical approach) was used to compare the mean DHT concentration, ovarian stromal cells areas, adipocyte diameters, mRNA levels, lipid concentration, TG concentration, and TC concentration between both groups and Fisher’s exact test (a non-parametric statistical approach) was used to compare the rates of irregular cycles between both groups in SPSS 21.0 package for Windows. *P*-values < 0.05 were considered significant.

## Results

### PCOS-Like Phenotypes in DHT-Treated Mice

**FIGURE 1:**
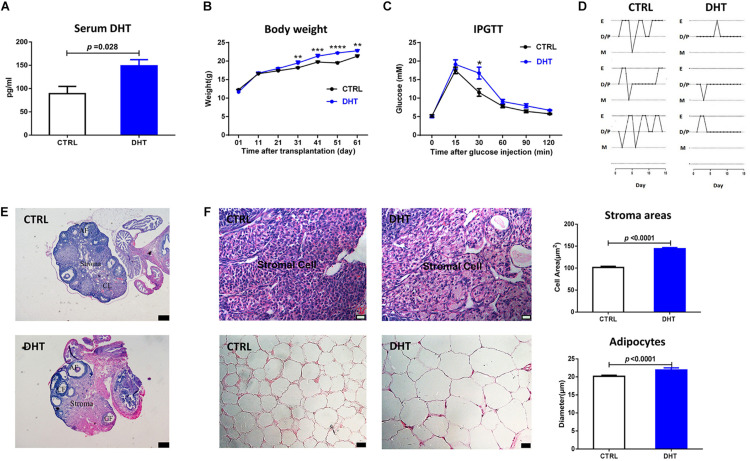
PCOS-like characteristics in DHT-treated mice. **(A)** Serum DHT level. **(B)** Body weight changes in mice. **(C)** Blood glucose changes in the IPGTT. **(D)** Estrous cycles. **(E)** Typical histological image of hematoxylin and eosin (H&E)-stained ovaries (corpora lutea, CL, cystic follicle, CF, antral follicle, AF, scale bars = 200 μm). **(F)** Morphology and mean size of ovarian stroma areas and adipocytes; *n* = 6. mean ± S.E.M. Mann-Whitney *U*-test for **(A,F)**, Duncan’s multiple range test for individual comparison repeated measures ANOVA test for **(B,C)**, **p < 0.01, ***p < 0.001, ****p < 0.0001.

To demonstrate the effects of DHT, parameters representing reproduction and metabolism were measured. The DHT levels in DHT-treated mice were significantly increased compared with those in placebo-treated mice (Figure 1A). In the late stage of the treatment, DHT-treated mice had higher body weights than placebo-treated mice (Figure 1B). The blood glucose levels of DHT-treated mice were higher than those of placebo-treated mice at 30 min after i.p. glucose injection (Figure 1C). DHT-treated mice had fewer regular cycles of 3–5 days than placebo-treated mice (Supplementary Figure 1A), resulting from prolong and stagnant estrus cycle, suggesting that the DHT-treated mice were acyclic (Figure 1D).

### Cell Hypertrophy and Lipid Accumulation in DHT-Treated Mice

The ovaries of DHT-treated mice showed no corpora lutea but some cyst-like antral follicles (Figure 1E and Supplementary Figure 1B). The stromal area and the diameters of the adipocytes was larger in DHT-treated mice than in placebo-treated mice (Figure 1F and Supplementary Figures 3, 4). These results suggest that DHT may induce the accumulation of lipid droplets in the stroma of ovaries and adipocytes of gonadal fat pads.

### Changed mRNAs Associated With Lipid Metabolism in the Ovaries and Gonadal Fat Pads of DHT-Treated Mice

**FIGURE 2:**
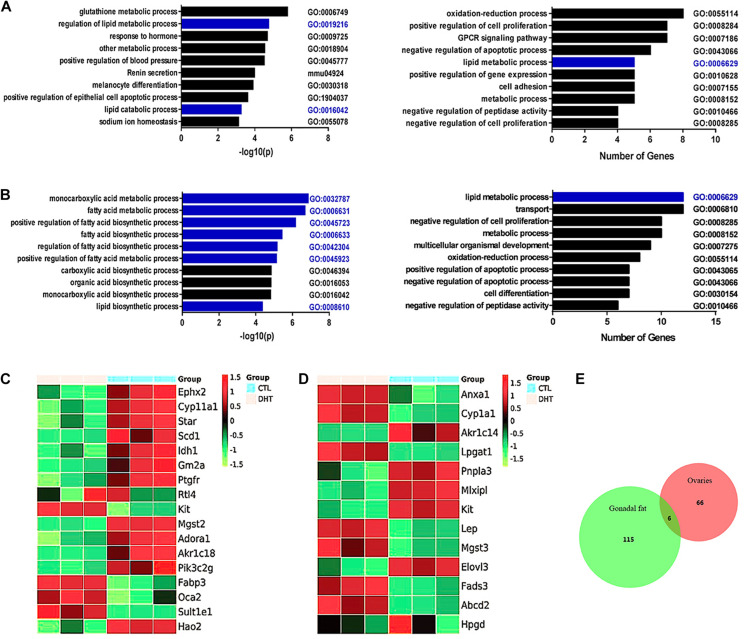
Gene expression analysis of ovaries and gonadal fats from ctrl and DHT treated mice. **(A)** Gene ontology (GO) term enrichment analysis of altered mRNAs in ovaries. Left panel show the top 10 biology processes based on the statistical significant for altered mRNAs. Right panel show the top 10 biology processes based on the amount of altered mRNAs; *n* = 3. **(B)** Gene ontology (GO) term enrichment analysis of altered mRNAs in gonadal adipose tissues. Left panel show the top 10 biology processes based on the statistical significant for altered mRNAs. Right panel show the top 10 biology processes based on the amount of altered mRNAs; *n* = 3. **(C)** Altered mRNAs associated with lipid metabolic processes in ovaries (Up-regulated, red, Down-regulated, green, *n* = 3). **(D)** Altered mRNAs associated with lipid metabolic processes in gonadal adipose tissues (Up-regulated, red, Down-regulated, green, *n* = 3). **(E)** Venn diagram shows the aggregation relationship between ovaries and adipose tissues with abnormal expression of genes affected by DHT. Overlapping region include *Col6a5*; *n* = 3.

The enriched gene ontology (GO) terms comprised 114 bioprocesses in ovaries. The top 10 enriched terms sorted in descending order based on −log(*P*)-values referred to the regulation of lipid metabolic processes (GO: 0019216) and lipid catabolic processes (GO: 0016042) (Figure 2A, left). The top 10 enriched terms sorted in descending order based on the numbers of changed genes also included lipid metabolic processes (GO: 0006629) (Figure 2A, right). Gene enrichment analysis also identified a significant decrease in metabolic processes including fatty-acyl-CoA metabolic process, long-chain-fatty-acyl- CoA metabolic process, neutral-lipid metabolic process, very-long-chain-fatty-acid metabolic process, and membrane-lipid catabolic process (Supplementary Figure 5).

The enriched GO terms contained 99 bioprocesses in gonadal fat pads. The top 10 enriched terms sorted based on − log(*P*)-values referred to the regulation of monocarboxylic acid metabolic processes (GO: 0032787), fatty acid metabolic processes (GO: 0006631), the positive regulation of fatty acid biosynthetic processes (GO: 0045723), fatty acid biosynthetic processes (GO: 0006633), the regulation of fatty acid biosynthetic processes (GO: 0042304), the positive regulation of fatty acid metabolic processes (GO: 0045923), and lipid biosynthetic processes (GO: 0008610) (Figure 2B, left). It is worth emphasizing that when based on the number of changed genes, the lipid metabolic process was the top enriched term (GO: 0006629) (Figure 2B, right). Gene enrichment analysis also identified a significant decrease in fatty-acyl-CoA metabolic process, very-long-chain-fatty-acid metabolic process, long-chain-fatty-acyl-CoA metabolic process, neutral-lipid catabolic process, and increase in lipid import into cell (Supplementary Figure 5).

Genes associated with lipid metabolic processes that were altered in DHT-treated mouse ovaries were selected and analyzed (Figure 2C). Compared with placebo-treated mice, DHT-treated mice showed decreased expression of *Adora1, Ephx2, Idh1, Scd1, StAR, Akr1c18, Cyp11a1, Hao2, Gm2a, Pik3c2g, Ptgfr*, and *Mgst2* and increased expression of *Fabp3, Kit, Oca2* and *Sult1e1*.

Altered genes associated with lipid metabolism processes in the gonadal fat pads of DHT-treated mice were selected and analyzed (Figure 2D). Compared with placebo-treated mice, DHT-treated mice showed decreased expression of *Elovl3, Kit, Mlxipl, Akr1c14*, and *Pnpla3* and increased expression of *Cyp1a1, Hpgd, Lep, Anxa1, Abcd2, Fads3, Lpgat1*, and *Mgst3*.

Gene expression was analyzed via the transcriptome array. According to the stricter selection criteria [fold-change ≥ 2, p ≤ 0.05 and false discovery rate (FDR) ≤ 10%], 66 mRNAs in the ovaries (Figure 2E, red circle) and 115 mRNAs in the gonadal fat pads (Figure 2E, green circle) were dysregulated in DHT-treated mice compared with placebo-treated mice. When comparing the dysregulated mRNAs in the ovaries and gonadal fat pads, we found that there were only six overlapping genes: *Col6a5*, *Ptgr*, *Ptgr1*, *Kit*, *Sult1e1, and C7* (Figure 2E).

### Inhibition of *Col6a5* Expression Alleviated the Hypertrophy of Ovaries and Gonadal Fat Pads in DHT-Treated Mice

**FIGURE 3:**
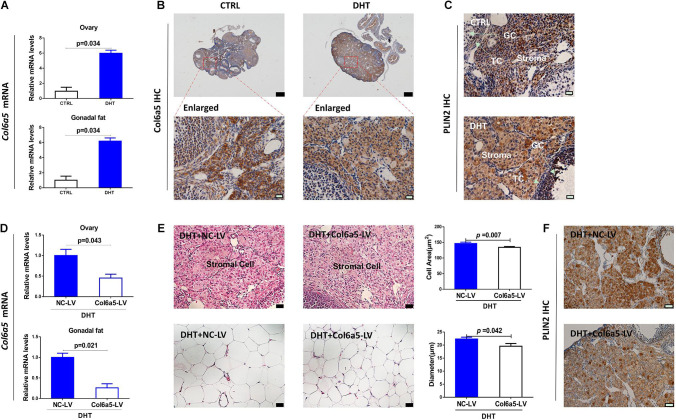
Inhibition of *Col6a5* expression alleviated the hypertrophy of ovaries and gonadal fat pads in DHT-treated mice. **(A)** Relative expression of *Col6a5* mRNA in the ovaries and gonadal fat pads showing a significant increase in *Col6a5* expression after DHT treatment; *n* = 6. The data are expressed as the mean ± SEM and were compared by Mann-Whitney *U*-test. **(B)** Immunohistochemistry of *Col6a5* in the ovaries showing a significant positive signal in ovarian stromal cells; *n* = 3, top, scale bars = 200 μm; bottom, scale bars = 20 μm. **(C)** Immunohistochemistry of PLIN2 (a marker of lipid droplets) in the ovaries showing a significant positive signal in hypertrophic ovarian stromal cells (GC, granulosa cells, TC, thecal cells); *n* = 3, scale bars = 20 μm. **(D)** Relative expression of *Col6a5* mRNA in the ovaries and gonadal fat pads was decreased in DHT- treated mice after *Col6a5*-LV injection; *n* = 6. The data are expressed as the mean ± SEM and were compared by Mann-Whitney *U*-test. **(E)** Histological images and mean size of ovarian stromal cells and gonadal adipocytes showing enhanced hypertrophy in DHT-treated mice after *Col6a5*-LV injection, the cell area is based on the same measurement as in Figure 1F; *n* = 6, scale bars = 20 μm. The data are expressed as the mean ± SEM and were compared by Mann-Whitney *U*-test. **(F)** Immunohistochemistry of PLIN2 (a marker of lipid droplets) in the ovaries showing decreased accumulation of lipid droplets in DHT-treated mice after *Col6a5*-LV injection; *n* = 3, scale bars = 20 μm.

Our verification showed that *Col6a5* was highly expressed in both the ovaries and gonadal fat pads of DHT-treated mice compared with placebo-treated mice (Figure 3A), which was consistent with previous mRNA array results. Additionally, IHC staining showed that *Col6a5* was expressed mainly in ovarian stromal cells rather than theca cells and granulosa cells (Figure 3B and Supplementary Figure 7). From the above, we suggest that *Col6a5* may be an important factor during the effect of DHT on ovarian stromal cells and adipocytes.

To test our hypothesis, we used *Col6a5*-CRISPR/Cas9 recombinant lentivirus to knockdown *Col6a5* expression in mice by tail vein injection. In the mice treated with DHT and injected with *Col6a5*-LV, the *Col6a5* mRNA levels were significantly decreased in both the ovaries and gonadal fat pads (Figure 3D). Compared with the NC-LV group, *Col6a5*-LV ameliorated the hypertrophy of ovarian stromal cells and adipocytes in DHT-treated mice (Figure 3E and Supplementary Figures 3, 4). The hypertrophy of ovarian stromal cells in DHT-treated mice was associated with excess lipid accumulation, as indicated by the expression of the lipid droplet-specific marker adipose differentiation-related protein adipophilin/perilipin-2 (PLIN2). From the immunochemistry results for PLIN2, we found that DHT treatment increased the accumulation of lipid droplets in ovarian stroma (Figure 3C) and this effect could be alleviated by the inhibition of *Col6a5* expression (Figure 3F). In addition, PLIN2 was also expressed in granulosa cells of antral follicles, especially after DHT treatment, this effect could also be alleviated by the inhibition of *Col6a5* expression, which is consistent with the results in stromal cells (Figures 3C,F).

### Inhibition of *Col6a5* Expression Alleviated DHT-Induced Excess Lipid Accumulation and Abnormal Steroidogenesis in Ovarian Stromal Cells

**FIGURE 4:**
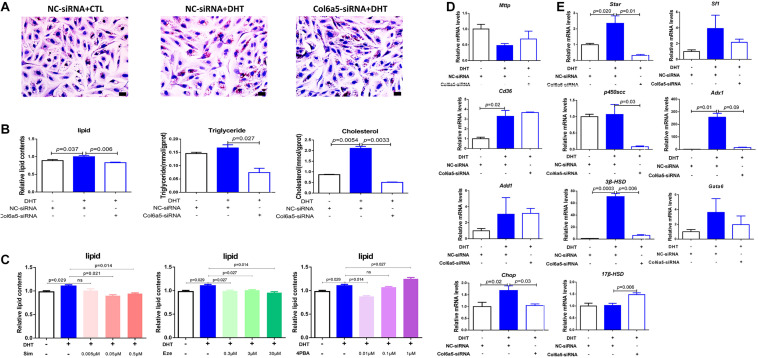
*Col6a5*-siRNA alleviated excess lipid accumulation in DHT-treated ovarian stromal cells. **(A)** Oil Red O staining of ovarian stromal cells; *n* = 3, Scale bars = 20 μm, NC-siRNA, negative control siRNA; *Col6a5*-siRNA, targeted *Col6a5* mRNA siRNA. Cells were cultured in medium with 1 μM DHT for 24 h. siRNA transfection was completed before DHT treatment. **(B)** Quantification of lipid, triglyceride and cholesterol levels in stromal cells. The lipid concentrations were calculated relative to the total protein concentration; *n* = 3. The data are expressed as the mean ± SEM and were compared by Mann-Whitney *U*-test. **(C)** Process-specific inhibition of cholesterol metabolism and relative quantification of lipid concentration in stromal cells. Simvastatin (Sim), an inhibitor of cholesterol synthesis; ezetimibe (Eze), an inhibitor of cholesterol uptake; and 4-PBA, an inhibitor of endoplasmic reticulum stress; *n* = 3. The data are expressed as the mean ± SEM and were compared by Mann-Whitney *U*-test. **(D)** Relative expression of lipid metabolism-related genes in stromal cells from each treatment group; *n* = 3. The data are expressed as the mean ± SEM and were compared by Mann-Whitney *U*-test. **(E)** Relative expression of cholesterol metabolism-related genes in interstitial cells from each treatment group; *n* = 3. The data are expressed as the mean ± SEM and were compared by Mann-Whitney *U*-test.

Oil Red O staining was used to identify and quantify oil droplets. DHT treatment significantly increased the formation of oil droplets in ovarian stromal cells at 1 μM. However, *Col6a5*-siRNA treatment relieved the effect of DHT and dramatically reduced lipid accumulation in the cells (Figure 4A).

The intracellular TG and TC levels were also quantified. DHT significantly increased the TC content in stromal cells compared with that in those not treated with DHT. However, both the TG and TC levels were significantly decreased in response to *Col6a5*-siRNA transfection of DHT-treated stromal cells (Figure 4B).

In addition, the TC content of stromal cells was much higher than the TG content, suggesting that TC may be the main component of the lipids that accumulate in response to DHT. Consistent with this, various concentrations of simvastatin (an inhibitor of cholesterol synthesis) and ezetimibe (an inhibitor of cholesterol uptake) were found to reduce the intracellular lipid content of DHT-treated stromal cells. Endoplasmic reticulum stress can induce cholesterol metabolism disorder, and 4-PBA (an inhibitor of endoplasmic reticulum stress) also reduced the lipid content of DHT-treated cells (Figure 4C).

We further investigated whether DHT affects the expression of lipid metabolism-related genes in stromal cells. *Mttp* promotes lipid transfer to the extracellular space; *Cd36* promotes lipid transfer to the intracellular space; *Add1* and *Chop* promote fat synthesis and lipid accumulation; *StAR* is a limiting enzyme of cholesterol mobilization; *P450scc* induces progesterone biogenesis; *3*β*-HSD* and *17*β*-HSD* promote testosterone biogenesis; *Sf1* promotes *StAR* expression; *Adx1* inhibits *Sf1* action; and *Gata6* promotes *P450scc* and *3*β*-HSD* expression.

We found that DHT led to higher *Cd36* and *Chop* mRNA levels than placebo treatment, while *Col6a5*-siRNA significantly suppressed the increase in *Chop* (Figure 4D). However, DHT significantly induced the expression of *StAR*, *3*β*-HSD*, and their transcription factor *Adx1*. Compared with the control siRNA (NC-siRNA), *Col6a5*-siRNA significantly suppressed the change in the expression of these genes in DHT-treated cells. In addition, the expression levels of *P450scc* were decreased, and the expression of *17*β*-HSD* was increased by *Col6a5*-siRNA in the DHT-treated cells, although DHT treatment alone did not alter the levels of these factors (Figure 4E).

### Inhibition of *Col6a5* Expression Alleviated DHT-Induced Excess Lipid Accumulation in Adipogenic 3T3-L1 Cells

**FIGURE 5:**
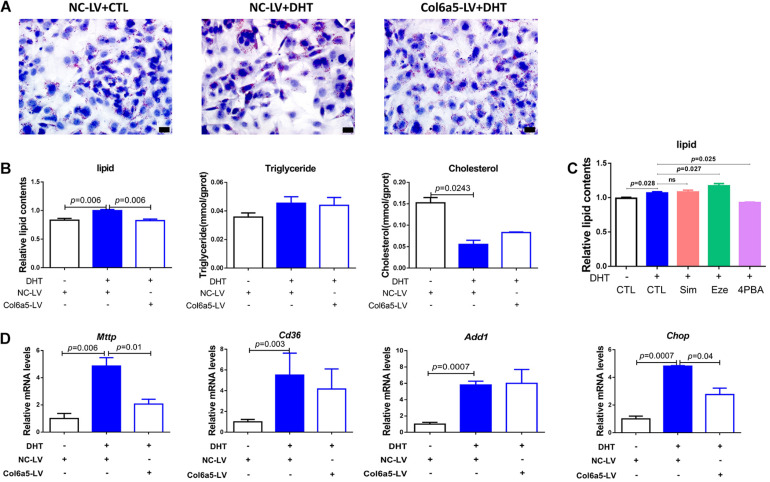
*Col6a5*-LV alleviated excess lipid accumulation in DHT-treated adipogenic 3T3-L1 cells. **(A)** Oil Red O staining of differentiated 3T3-L1 cells; *n* = 3; scale bars = 20 μm; NC-LV, negative control lentivirus; *Col6a5*-LV, LV-*Col6a5*-CRISPR/Cas9 recombinant lentivirus. Cells were cultured in adipogenic differentiation cocktail media with 1 μM DHT. Lentivirus transfection was completed before DHT treatment. **(B)** Quantification of lipid, TG and TC levels in differentiated 3T3-L1 cells; *n* = 3. The data are expressed as the mean ± SEM and were compared by Mann-Whitney *U*-test. **(C)** Process-specific inhibition of cholesterol metabolism and relative quantification of lipid concentration in differentiated 3T3-L1 cells from each treatment group; *n* = 3. The data are expressed as the mean ± SEM and were compared by Mann-Whitney *U*-test. **(D)** Relative expression of lipid metabolism-related genes in differentiated 3T3-L1 cells from each treatment group; *n* = 3. The data are expressed as the mean ± SEM and were compared by Mann-Whitney *U*-test.

DHT treatment at 1 μM significantly increased the accumulation of oil droplets in adipogenic 3T3-L1 cells. However, this effect was dramatically alleviated after transfection with *Col6a5*-LV (Figure 5A). The TC content was decreased after treatment with DHT, while there was no significant difference in 3T3-L1 cells transfected with *Col6a5*-LV (Figure 5B).

While the level of TC was higher than that of TG in stromal cells, the TC content was similar to the TG content in adipogenic 3T3-L1 cells. Moreover, simvastatin and ezetimibe could not suppress the excess lipid accumulation induced by DHT, suggesting that TC may not be the main component of the lipids that accumulate in response to DHT. However, 4-PBA reduced the intracellular lipid content in DHT-treated adipogenic 3T3-L1 cells (Figure 5C). This implies that DHT may dysregulate lipid metabolism in adipocytes by inducing endoplasmic reticulum stress.

DHT also influenced the expression of lipid metabolism-related genes in adipogenic 3T3-L1 cells. As shown in Figure 5D, DHT significantly induced the expression of *Mttp, Cd36, Add1*, and *Chop*, and the increases in the expression of *Mttp* and *Chop* were significantly attenuated after transfection with *Col6a5*-LV (Figure 5D).

### Inhibition of *Col6a5* Expression Alleviated Overnutrition-Induced Excess Lipid Accumulation in NCTC1469 Mouse Hepatic Cells

**FIGURE 6:**
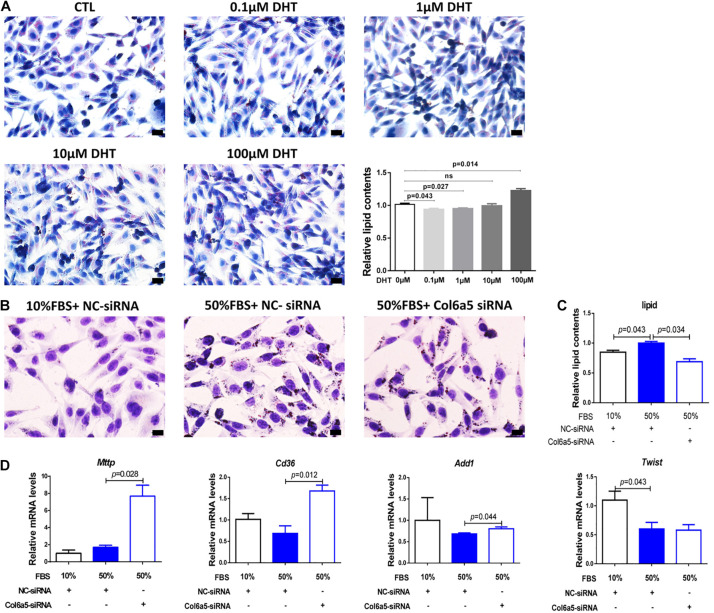
*Col6a5*-siRNA alleviated excess lipid accumulation in overnourished NCTC1469 cells. **(A)** Oil Red O staining and relative quantification of lipid concentrations in DHT-treated NCTC1469 cells; *n* = 3, scale bars = 20 μm. NCTC1469, a mouse hepatic cell line. Cells were cultured in medium with DHT for 24 h; *n* = 3. The data are expressed as the mean ± SEM and were compared by Mann-Whitney *U*-test. **(B)** Oil Red O staining and **(C)** relative quantification of lipid concentrations in overnourished NCTC1469 cells; *n* = 3, scale bars = 20 μm. **(D)** Relative expression of lipid metabolism-related genes in NCTC1469 cells from each treatment group; *n* = 3. The data are expressed as the mean ± SEM and were compared by Mann-Whitney *U*-test.

The effect of DHT on lipid accumulation was cell type-specific, since there was no increase in lipid droplets in the DHT-treated NCTC1469 cells at a dose similar to that used to treat stromal cells and 3T3-L1 cells (Figure 6A). As an overnourished cell model, NCTC1469 cells exhibited an increase in lipid droplets when cultured in 50% FBS (v/v) medium, and *Col6a5*-siRNA relieved this effect (Figure 6B). Inhibition of *Col6a5* expression commonly affected intracellular lipid accumulation (Figure 6C). However, 50% FBS treatment did not significantly alter the expression of *Mttp, Cd36* and *Add1*, the expression of *Twist2*, a transcription factor of *Add1*, was even decreased, while *Col6a5* siRNA could significantly increase the expression of *Mttp, Cd36* and *Add1* (Figure 6D).

## Discussion

Hyperandrogenism is a key trait of PCOS (Nisenblat and Norman, 2009), and excess androgen experimentally induces a range of PCOS-like phenotypes, including reproductive and metabolic phenotypes, in various animal models (Walters et al., 2012; Abbott et al., 2013). There have been many reports on the characteristics of the ovaries and adipose tissue of DHT-treated mice. However, these reports present limited descriptions and analyses of the alterations in gene expression and bioprocess pathways in reproductive- and metabolism-related tissues affected by DHT treatment. Additionally, the tissue-specific mechanisms through which androgens elicit these phenotypes remain unclear. Hence, in this study, we analyzed the gene expression profiles of DHT-treated mouse ovaries and gonadal fat pads via RNA microarray analysis, focusing on the molecular mechanism by which DHT alters metabolic processes.

Data on the gene expression profiles of rats have been published. Bioinformatics analyses of microarray data from DHT-treated rat ovaries have shown that DHT influences ovarian lipid metabolism and cell proliferation (Hossain et al., 2013; Salilew-Wondim et al., 2015), which was consistent with our data. Additionally, our data included an analysis of total mRNA and related bioprocess alterations in both the ovaries and gonadal fat pads of DHT-treated mice, offering a more comprehensive analysis.

From morphological observations, we found that mice treated with DHT display hyperplasia of ovarian stroma and enlarged of gonadal adipocytes. Meanwhile the microarray data indicated that the expression of genes and the functions of bioprocesses related to lipid metabolism were altered in both the ovaries and gonadal fat pads of DHT-treated mice, indicating that excess lipid accumulation may have occurred in these cells. However, the features of lipid metabolic disorder associated with hyperandrogenism were tissue-specific. In the ovaries, genes associated with lipid metabolic processes, including *Wnt10b, Cyp11a1, Scd1, Ephx2*, and *Gm2a*, were altered by DHT; in the gonadal fat pads, *Ptges, Hpgd, Fads3*, and *Elovl3* were altered. Interestingly, although there are many dysregulated genes in the ovaries (66 mRNAs) and gonadal fat pads (115 mRNAs) of mice treated with DHT, there were only six overlapping genes: *Col6a5*, *Ptgr*, *Ptgr1*, *Kit*, *Sult1e1*, and *C7.*

DHT induced excess lipid accumulation in stromal cells, significantly altered cholesterol metabolism, increased cholesterol levels and led to the abnormal expression of genes associated with cholesterol metabolism and steroidogenesis. It is worth noting that DHT can also induce the accumulation of lipid droplets in granulosa cells of antral follicles, which may be one of the reasons for DHT leads to abnormal steroidogenesis in ovary (Tang et al., 2016). In addition, we found that *Col6a5* was expressed in granulosa cells of ovarian antral follicles, luteal cells and stroma at different stages of superovulation (Supplementary Figure 7). Since the development of follicle and corpus luteum is accompanied by the synthesis and secretion of many steroid hormones, including estrogen and progesterone (Yang et al., 2016), we believe that *Col6a5* may also be involved in steroid hormone synthesis. Therefore, by inhibiting the expression of *Col6a5 in vitro*, we verified that the abnormal lipid metabolism and steroidogenesis induced by DHT were improved.

The mechanism of DHT induced excess lipid accumulation in adipocytes seems to be different from that of stromal cells, probably by affecting endoplasmic reticulum stress rather than cholesterol metabolism, although it can also be alleviated by inhibiting the expression of *Col6a5* both *in vivo* and *in vitro*.

NCTC1469 murine hepatic cells treated with DHT at normal concentration did not accumulate lipid droplets, which indicated that the effect of DHT was cell type-specific. However, *in vitro* inhibition of *Col6a5* expression could still alleviate FBS-induced lipid accumulation in NCTC1469. 50% FBS treatment did not significantly alter the expression of lipid-related genes, indicating that the way of lipid accumulation induced by overnutrition is different from that induced by DHT, while *Col6a5* siRNA can significantly increase the expression of lipid-related genes, which may be related to cell specificity and needs further study to clarify.

**FIGURE 7:**
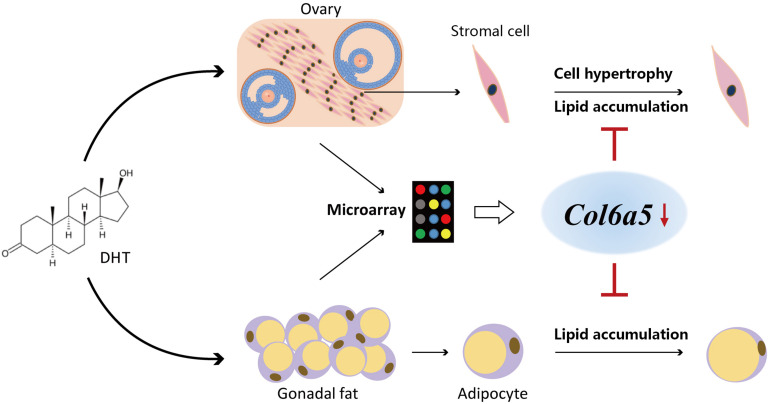
Inhibition of *Col6a5* attenuates excessive lipid accumulation induced by DHT. DHT acts directly on ovarian and gonad fat cells, leading to lipid metabolism disorder, which in turn leads to excess lipid accumulation and cell hypertrophy. Inhibition of the expression of our screened target gene *Col6a5* could alleviate DHT-induced excess lipid accumulation, hypertrophy and abnormal metabolism-related gene expression.

In summary, our present findings show that DHT acts directly on ovarian and gonad fat cells, leading to lipid metabolism disorder, which in turn leads to excess lipid accumulation and cell hypertrophy. However, inhibition of the expression of our screened target gene *Col6a5* could alleviate DHT-induced excess lipid accumulation, hypertrophy and abnormal metabolism-related gene expression (Figure 7). These results indicate that the *Col6a5*-mediated lipid metabolism pathway is involved in hyperandrogenic pathologies and may be utilized for the development of novel therapeutics.

## Data Availability Statement

The datasets presented in this study can be found in online repositories. The names of the repository/repositories and accession number(s) can be found below: NCBI GEO (Accession: GSE171431).

## Ethics Statement

The Committee on the Use of Live Animals for Teaching and Research, Shenzhen Institutes of Advanced Technology, Chinese Academy of Sciences. All animal experimental methods were performed in accordance with the approved guidelines and regulations.

## Author Contributions

JZ and L-FS conceived and designed the experiments. JZ, P-GR, and CH contributed to supervision and funding acquisition. L-FS and Y-LY performed the experiments, analyzed the data, and wrote the manuscript. M-YW and H-SZ revised the manuscript. T-XX, M-XL, and B-BW contributed to the methodology, reagents, and laboratory equipment. All authors contributed to the article and approved the submitted version.

## Conflict of Interest

The authors declare that the research was conducted in the absence of any commercial or financial relationships that could be construed as a potential conflict of interest.

## Footnotes

1 https://www.gcbi.com.cn

2 http://www.metascape.org
