# Supplementary material for: Inhibition of Col6a5 Improve Lipid Metabolism Disorder in Dihydrotestosterone-Induced Hyperandrogenic Mice
Source: Front Cell Dev Biol. 2021 May 24;9:669189. doi: 10.3389/fcell.2021.669189 (PMC8181728; doi:10.3389/fcell.2021.669189)
Supplement: Supplementary file 1 [file Data_Sheet_1.docx]

Supplementary Material

# Supplementary Data

Supplementary Material should be uploaded separately on submission. Please include any supplementary data, figures and/or tables. All supplementary files are deposited to FigShare for permanent storage and receive a DOI.

Supplementary material is not typeset so please ensure that all information is clearly presented, the appropriate caption is included in the file and not in the manuscript, and that the style conforms to the rest of the article. To avoid discrepancies between the published article and the supplementary material, please do not add the title, author list, affiliations or correspondence in the supplementary files.

# Supplementary Figures and Tables

For more information on Supplementary Material and for details on the different file types accepted, please see [here](http://home.frontiersin.org/about/author-guidelines#SupplementaryMaterial). Figures, tables, and images will be published under a Creative Commons CC-BY licence and permission must be obtained for use of copyrighted material from other sources (including re-published/adapted/modified/partial figures and images from the internet). It is the responsibility of the authors to acquire the licenses, to follow any citation instructions requested by third-party rights holders, and cover any supplementary charges.

## Supplementary Figures


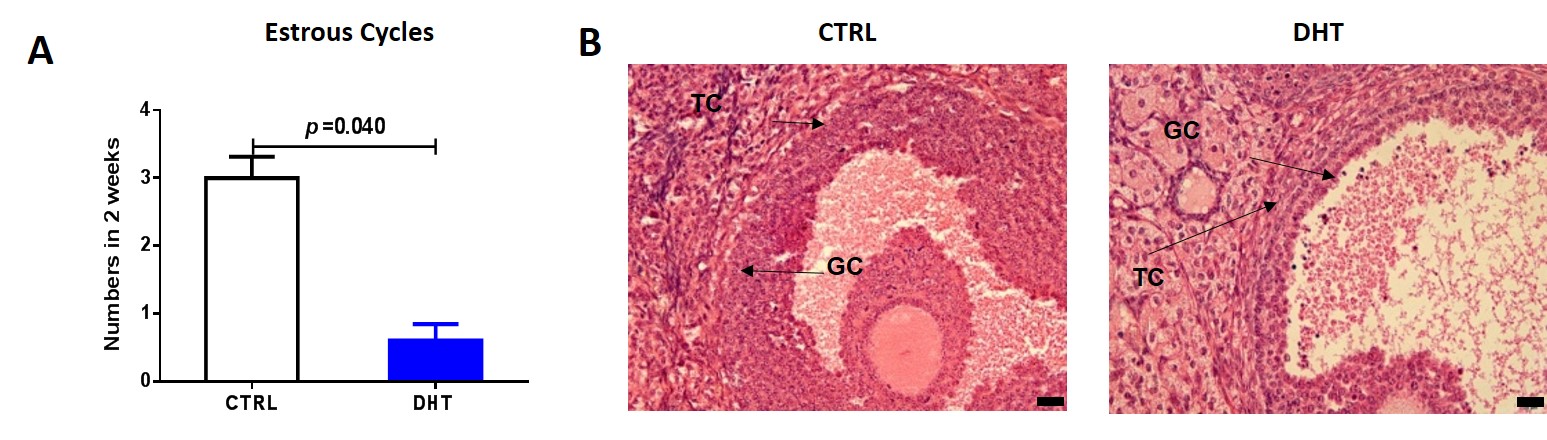


**Supplementary Figure 1.** (A) Estrous cycles. n = 6. The data are expressed as the mean ± SEM and were compared by Fisher’s exact test. (B) Morphology of ovaries. Thecal cells, TC, granulosa cells, Scale bars=20 μm.


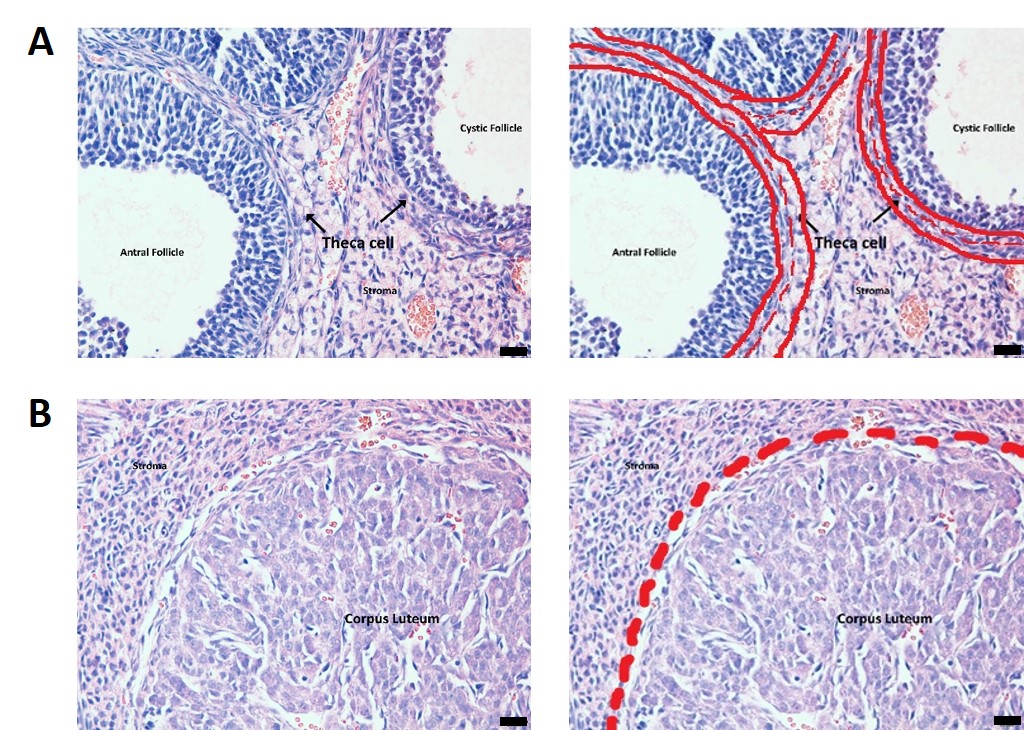


**Supplementary Figure 2.** (A) Outline of the theca cell area, Scale bars=20 μm. (B) Division of stromal area and corpus luteum, Scale bars=20 μm.


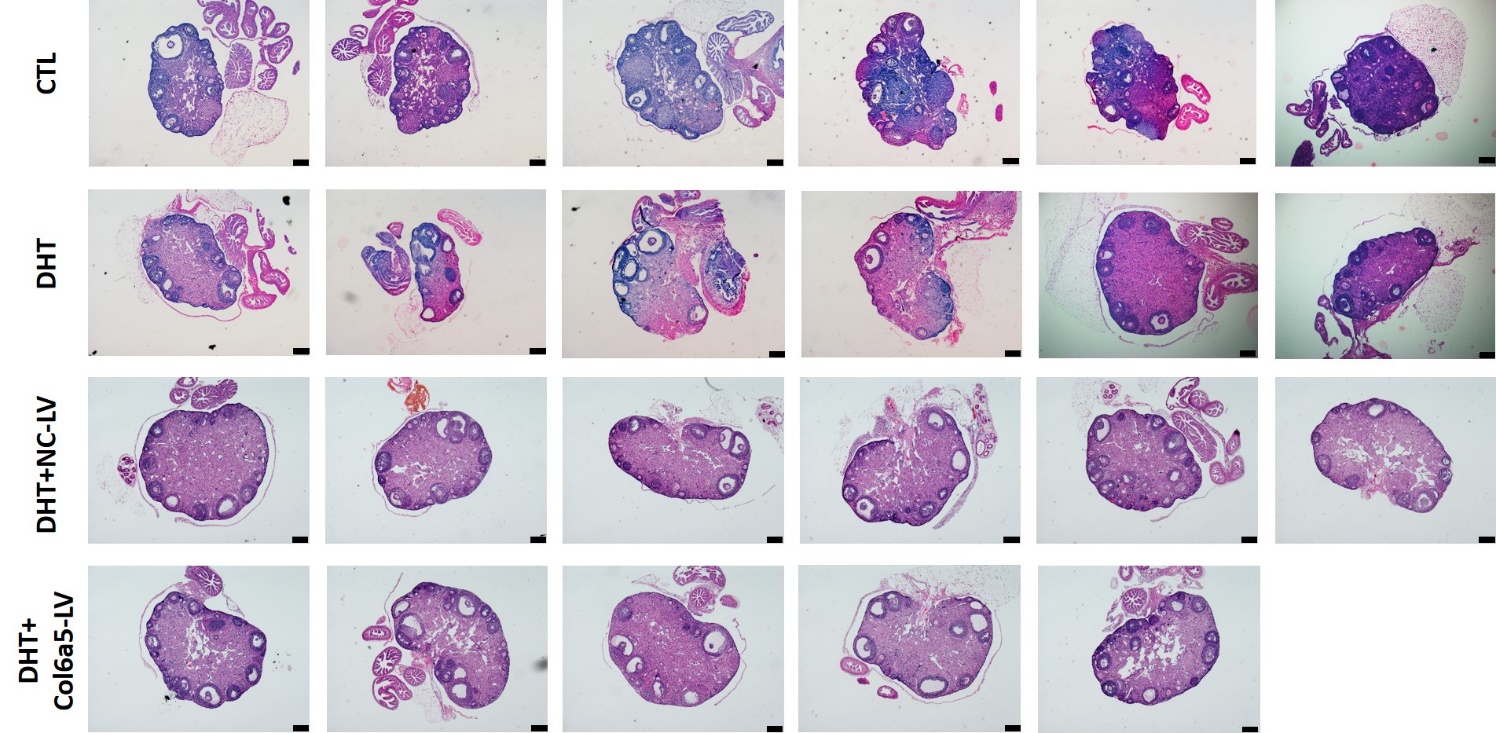


**Supplementary Figure 3.** Morphology of ovaries from each treatment group, bar=200 μm.


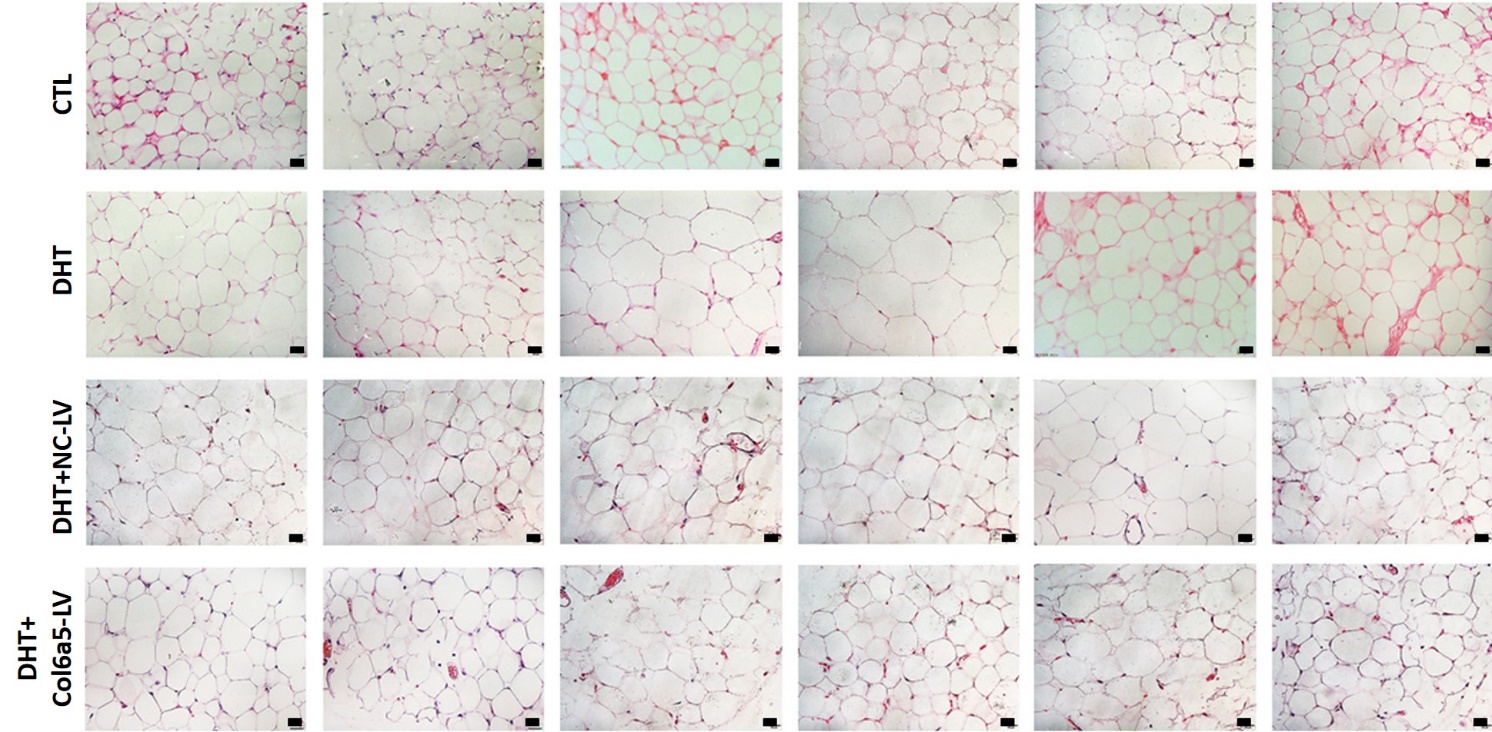


**Supplementary Figure 4.** Morphology of gonadal fat from each treatment group, bar=20 μm.


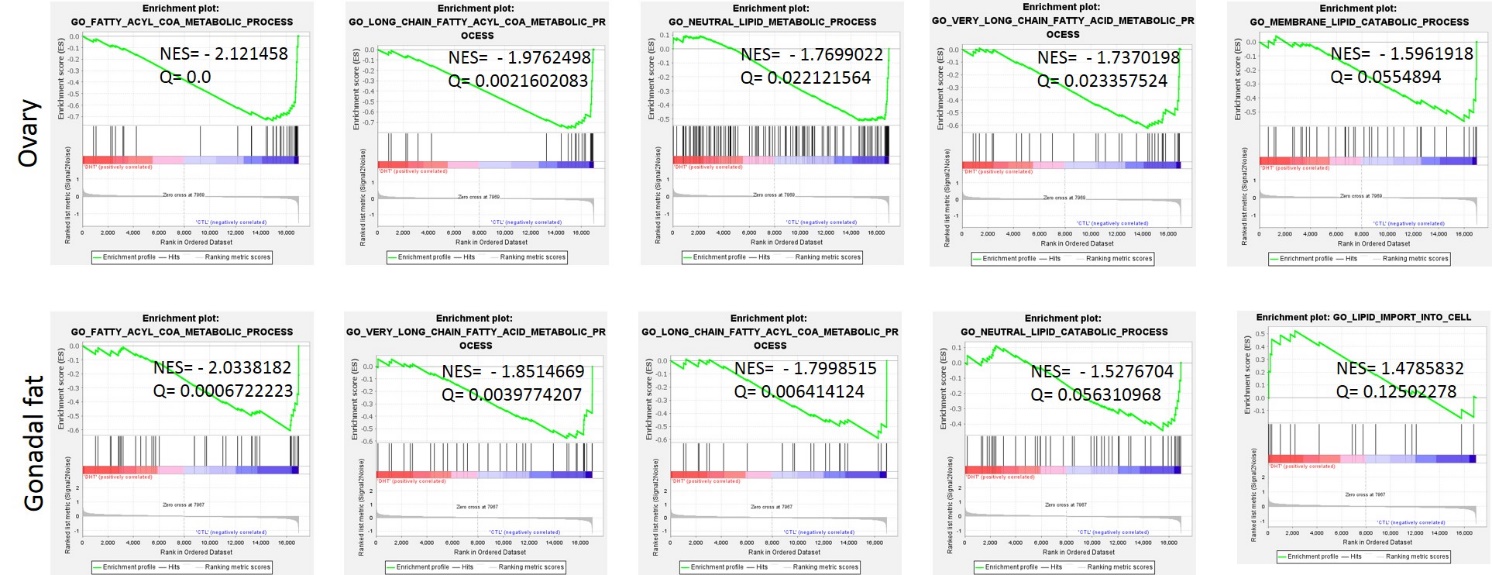


**Supplementary Figure 5.** GSEA analysis showed that the treatment of DHT decreased lipid metabolism and associated signatures in ovaries and gonadal fat tissues. Normalized enrichment score (NES) and false-discovery rate (FDR) Q value.


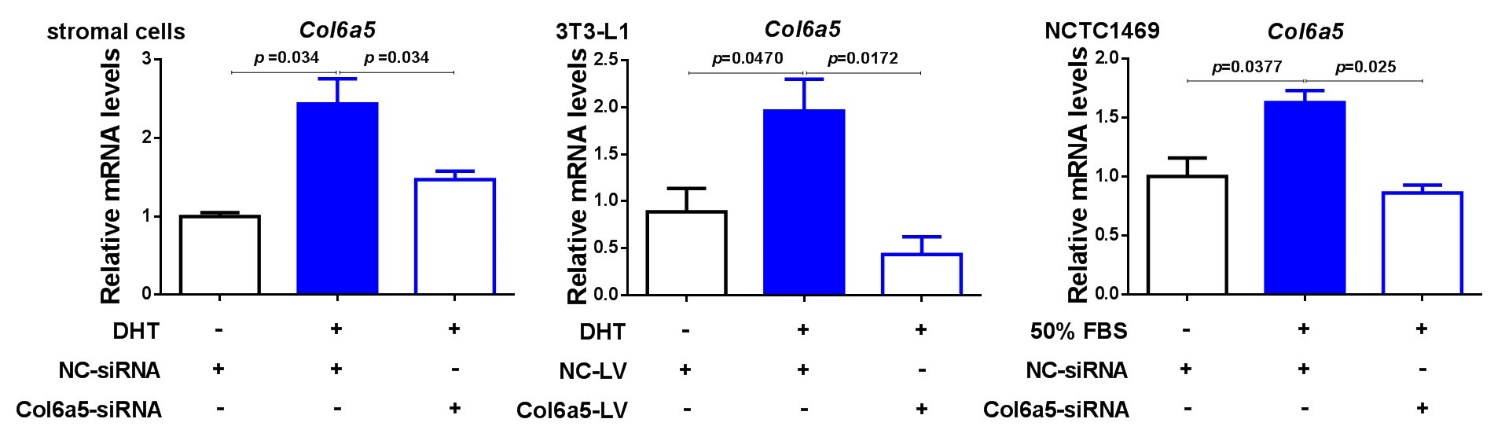


**Supplementary Figure 6.** Relative expression of *Col6a5* genes in ovarian stromal cells, 3T3-L1 and NCTC1469 cells from each treatment group. n = 3. The data are expressed as the mean ± SEM and were compared by Mann-Whitney *U*-test. NC-siRNA, negative control siRNA; *Col6a5*-siRNA, targeted *Col6a5* mRNA siRNA. Cells were cultured in medium with 1 μM DHT for 24 h. siRNA transfection was completed before DHT treatment; NC-LV, negative control lentivirus; *Col6a5*-LV, LV-*Col6a5*-CRISPR/Cas9 recombinant lentivirus. Cells were cultured in adipogenic differentiation cocktail media with 1 μM DHT. Lentivirus transfection was completed before DHT treatment.


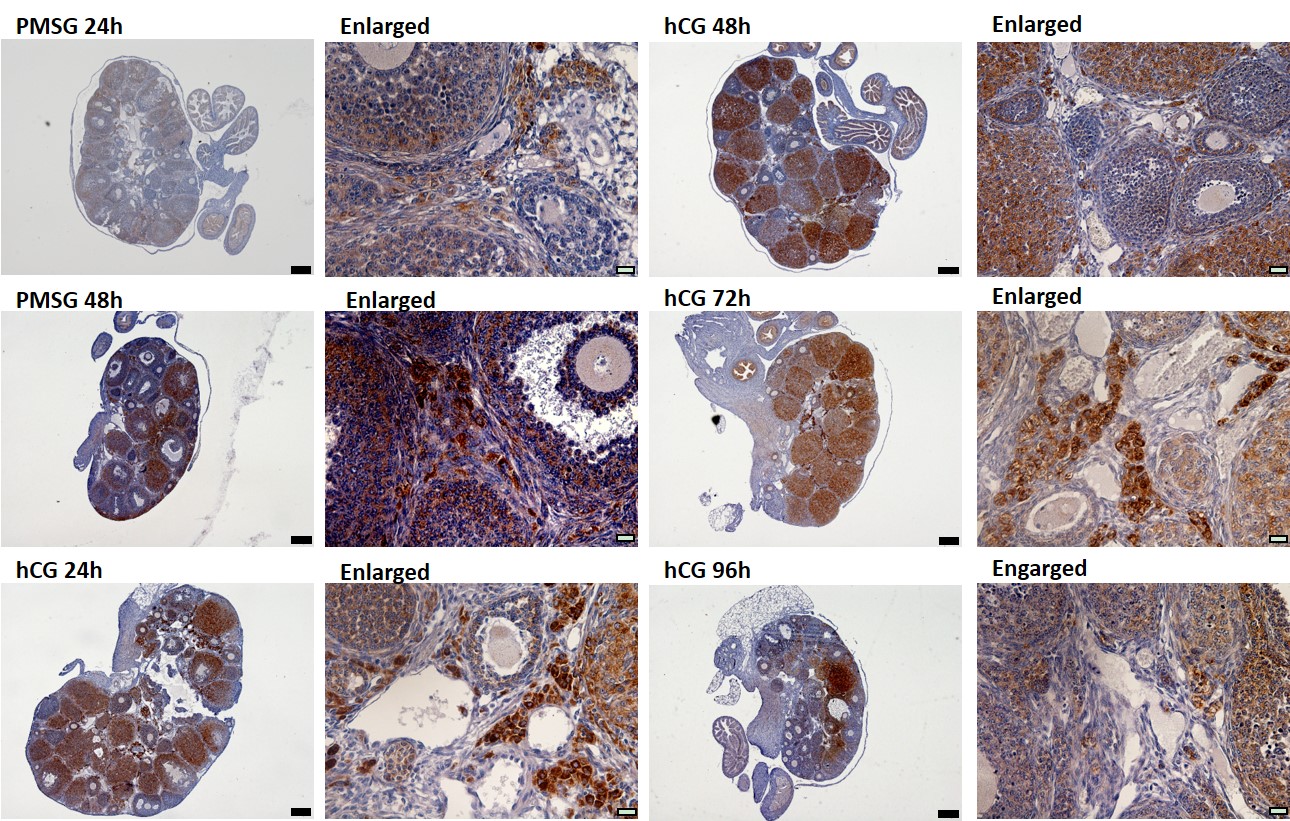


**Supplementary Figure 7.** Immunohistochemistry of Col6a5 in ovaries at different stages of superovulation, showing ubiquitous localization in ovarian stromal cells, but not in all granulosa cells and thecal cells. n = 6 per treatment group, bar=200 μm, enlarged, bar=20 μm.
